# The effect of treatment with a non-invasive foot worn biomechanical device on subjective and objective measures in patients with knee osteoarthritis- a retrospective analysis on a UK population

**DOI:** 10.1186/s12891-020-03382-3

**Published:** 2020-06-16

**Authors:** Christopher Miles, Andrew Greene

**Affiliations:** Sport and Exercise Science Research Centre, Department of Life Sciences, University of Roehampton, 19 Conifer drive, Brentwood, London, CM14 5TZ UK

**Keywords:** Knee osteoarthritis, Gait, Pain, Function, Biomechanical treatment

## Abstract

**Background:**

Osteoarthritis is a major cause of pain and disability worldwide, therefore ways of treating this condition are paramount to a successful health system. The purpose of the study was to investigate the changes in spatial-temporal gait parameters and clinical measurements following treatment with a non-invasive foot-worn biomechanical device on patients with knee osteoarthritis within the UK.

**Methods:**

A retrospective analysis was carried out on 455 patients with knee osteoarthritis. All patients were evaluated using a computerized gait test and two self-assessment questionnaires (WOMAC and SF-36) at baseline and after 3 and 6 months of treatment. The biomechanical device is a shoe-like device with convex pods under the sole that have the capability of changing foot centre of pressure and training neuromuscular control. The device was individually calibrated for each patient to minimise symptoms whilst walking and train neuromuscular control. Patients used the device for short periods during activities of daily living. Repeated measures statistical analyses were performed to compare differences over time.

**Results:**

After 6 months of treatment significant improvements were seen in all gait parameters (*p* < 0.01). Specifically, gait velocity, step length and single limb support of the more symptomatic knee improved by 13, 7.8 and 3%, respectively. These were supported by significant improvements in pain, function and quality of life (48.6, 45.7 and 22% respectively; *p* < 0.001). A sub-group analysis revealed no baseline differences between those who were recommended joint replacement and those who were not. Both groups improved significantly over time (*p* < 0.05 for all).

**Conclusions:**

Our results suggest that the personalised biomechanical treatment can improve gait patterns, pain, function and quality of life. It may provide an additional solution to managing UK patients suffering from knee osteoarthritis but needs to be tested in a controlled setting first.

## Background

Osteoarthritis (OA) is a major cause of pain and locomotor disability worldwide. The knee is the most commonly affected weight-bearing joint with 4.11 million people having knee OA in England [1]. With an aging population and a rise in obesity being leading risk factors [2], it is estimated that by 2020, the number of people suffering from knee OA will rise to 6.5 million [1]. The condition leads to social, psychological and economical burdens with substantial financial consequences [3]. Therefore, cost-effective and non-invasive ways of treating and managing this condition more effectively are paramount to a successful health system.

Currently, the National Institute of Clinical Excellence (NICE) guidelines outline core treatments such as education and exercise as first line care, progressing to more advanced biomechanical modalities such as valgus knee braces and orthotics in some cases [4]. Total knee replacement (TKR) is the most common treatment for end-stage knee OA and appears to be increasing over-time [5] with studies estimating that the rates of TKR will reach 119,000 procedures per year by 2035 [6]. TKR’s have revolutionised the care of patients with knee OA and are considered an effective intervention for the treatment of chronic knee pain and disability [7]. However, as well as the associated issues of surgical intervention, there is also evidence based on using patient reported outcome measures (PROM’s), that some patients experience chronic knee pain, functional disability, and poor quality of life after TKR [8]. It is suggested approximately 18% of patients report the outcomes of their surgery as only fair or poor, with a small proportion of these experiencing complications [9]. One plausible explanation is related to a poor patient selection process. Although the NHS is trying to optimise the selection criteria for TKR, ultimately it is a shared decision between the physician and the patient. There is currently no objective marker that accurately identifies the functional severity of a patient. In addition, patients following TKR often continue to have altered muscular activity, a possible contributor to symptoms remaining unaddressed.

One factor that has been heavily researched over the past few decades is the effect of lower limb biomechanics upon the development and progression of the disease [10, 11]. Patients with knee OA often present changes in spatial-temporal gait parameters including a reduction in gait velocity, step length and single limb support phases. Moreover, these changes were found to correlate with the levels of pain and functional disability [12, 13]. External knee adductor moment (KAM) and knee adductor angular impulse (KAAI) have been suggested as the surrogate kinetic variables for expressing medial joint forces and overall cumulative loading of the knee throughout the stance phase respectively [11, 14, 15]. Neuromuscular changes related to knee osteoarthritis such as muscle weakness and altered muscle activation have also been widely acknowledged as contributing factors to the development of OA [11, 16–19].

Symptomatic knee OA has been shown to be influenced by both biomechanical and neuromuscular treatments, which has led to conservative treatments currently available largely aiming to influence either one of these factors [20–27]. Interventions typically attempt to manipulate the KAM and KAAI loads transmitted through the joint, or improve the neuromuscular deficits seen within the pathology by muscle strengthening and proprioceptive exercises [28, 29]. However, these modalities often occur in isolation and can reduce effect in time, which may bring into question their effectiveness, leading to further deterioration of symptoms and eventually surgery [22, 30, 31].

**Fig. 1:**
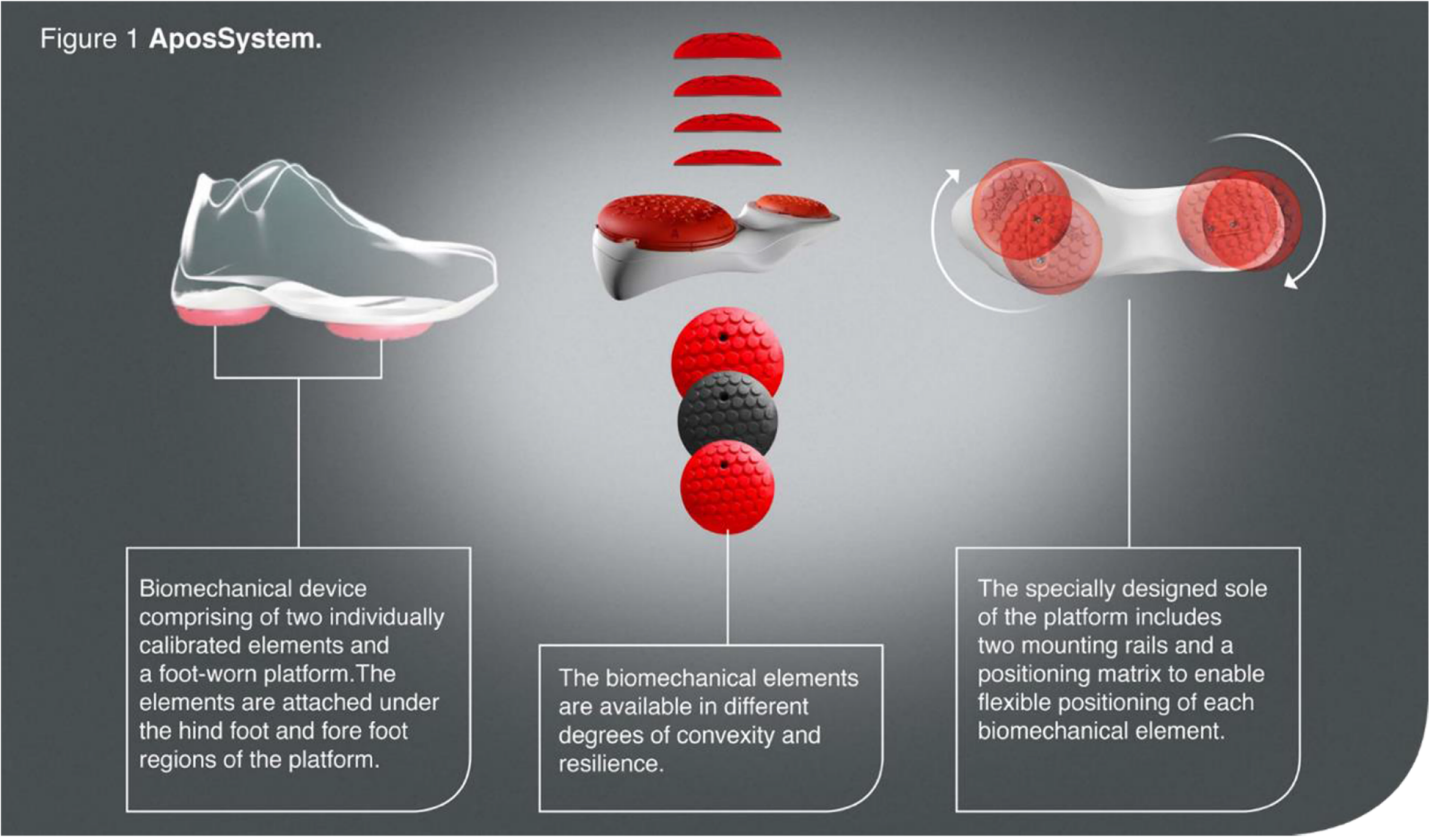
The biomechanical device. Image was provided by the company and was approved to be used

For the past decade, a personalised non-invasive biomechanical treatment for patients with knee OA has been available in the UK. In essence, it is a specially made shoe-like device which provides the platform to fit two convex pods under the sole. One is located under the anterior part of the sole and the other under the posterior, both attached using special rails and screws (Fig. 1). This foot-worn device has the capability of changing foot centre of pressure and training neuromuscular control. The biomechanical device is individually calibrated to each patient based on their gait patterns and clinical symptoms. The patient then receives a home-based treatment plan and is asked to return to the clinic for follow-up appointments to assess progress and re-adjust the pods calibration if needed.

There is extensive evidence about the effect of this treatment on clinical outcomes of patients with knee OA is promising [31–35], albeit no research is yet published on UK populations. Some cultural differences are associated with the prevalence of knee OA [36, 37] and although it is reasonable to assume that the clinical effect will be the same with regards to alleviation in symptoms [31, 34, 38], it is important to validate this treatment in new populations. Therefore, this study aimed to investigate the changes in spatial-temporal gait parameters, gait metrics of severity and clinical measurements (pain, function and quality of life) following treatment with a non-invasive foot-worn biomechanical device on patients with knee OA within the UK. We hypothesize that this treatment will have similar effect on the UK population as was described on other populations within previous studies [31–34].

## Methods

**Fig. 2:**
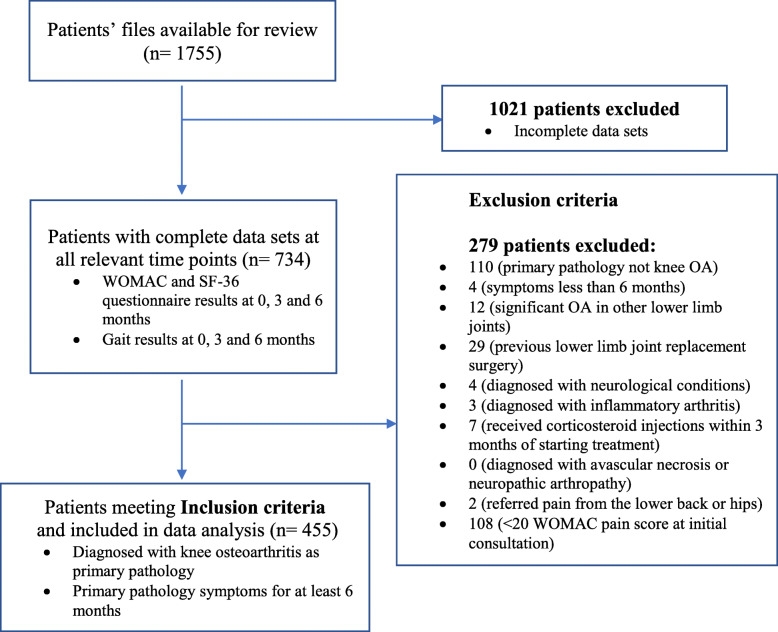
Flow chart of study screening and inclusion

A retrospective chart review analysis was carried out to examine the effect of the biomechanical treatment upon spatial-temporal gait parameters, levels of pain and function, along with perceptions of quality of life in patients suffering from knee OA. The protocol was approved by the University of Roehampton Ethics Committee and all participants provided consent for their data to be used within in the study. A search for eligible data was conducted on the companies’ database providing the biomechanical treatment between 2009 and 2017. The treatment has been available in the UK since 2009 via private clinics that were operated by the company. It is positioned to treat primary-care patients that have usually failed to respond to traditional modalities (physiotherapy/medications) that were looking for a solution to their MSK condition. Figure 2 represents the data reduction flow chart and the eligibility for the study against the inclusion/exclusion criteria.

Four hundred and fifty-five patients, 247 females (54%) and 208 males (46%) with symptomatic knee OA participated in this study. Mean (SD) age was 61.6 (8.7) years and 62.9 (10.3) years, respectively (Table 1). All patients completed both computerised gait analysis and validated PROM’s at initial consultation and at 3 and 6 months after commencing treatment. The OptoGait system (Version 1.11) was used by trained physiotherapists to measure spatial-temporal gait parameters at each data collection point [39]. It works by detecting the interruption between the transmitting and a receiving LED (light emitting diode) bars. This interruption produces quantifiable data that can highlight differences between normal to pathological gait such as in knee OA [13, 40]. Patients walked at a self-selected speed over a 4 m measurement area, with 2 m before and after to allow for constant velocity walking speed during data capture. Each gait test included 4 lengths in which the mean values were calculated for each parameter. The parameters recorded include velocity (cm/s), step length (cm) and single-limb support (SLS) phase (% gait cycle). Step length and SLS was calculated for the less and more symptomatic limbs respectively. For patients with bilateral OA, the more symptomatic limb was determined by the lower SLS at baseline. For patients with unilateral OA, the more symptomatic limb was the limb that was reported with OA. All gait assessments were conducted in barefoot at each data collection point and occurred within one of the biomechanical treatments’ clinics (London, Birmingham or Manchester).

**Table 1:** Patient Characteristics

Total patients:	455	
Male (% of total)	208 (46%)	
Female (% of total)	247 (54%)	
Age (years)	62.2 (9.5)	
Unilateral vs Bilateral knee OA (%)	204 (45%) vs 251 (55%)	
**Sub-group data**		
Group	Not recommended surgery	Recommended surgery
Number of patients (%)	363 (80%)	92 (20%)
Duration of symptoms (months)	67	83

The Knee Osteoarthritis Function Grade (KOFG) was used to classify patients’ and assess improvements in gait over the treatment period. It is a validated classification tool utilising cadence and stride length from the spatial-temporal gait analysis as predictor variables to assess changes in functional severity [13]. The KOFG is a four-grade scale with 1 being the best function and 4 the worst function. According to the model, a shorter stride length with lower cadence is indicative of a higher functional severity grade disease (more severe knee OA), while a longer stride length with higher cadence is indicative of a lower functional severity grade of disease (less severe knee OA).

The WOMAC questionnaire was used to evaluate changes in patients’ perception of pain and function [41]. It contains 24 visual analogue scale (VAS) questions that can be divided in 3 sub-categories (Pain: 5 Q, Functional Limitation: 17 Q, and Stiffness: 2 Q). Results range from 0 to 100 mm, in which 0 mm indicates no pain and 100 mm indicates the most severe pain or limitation in function. The SF-36 is a recognised quality of life questionnaire [42] and is scored between 0 and 100, with 0 indicating the worst quality of life and 100 indicating the best quality of life. The total score is made up of 8 sub-categories which make up 2 summarising scores; Physical Component Summary (PCS) and the Mental Component Summary (MCS). These reflect the patients’ physical/mental condition respectively.

After the completion of the baseline measurements, the biomechanical devices (Apos System, Apos Medical Assets Ltd. Tel-Aviv, Israel; Fig. 1) were individually calibrated to each patient by a physiotherapist certified in the treatment methodology. Patients wear a pair of devices regardless of whether bilateral or unilateral symptoms. The principle of the calibration is to reduce pain in the knee during walking. From a biomechanical perspective, shifting the elements on the shoe changes the foot’s centre of pressure (COP) during gait with the goal to re-orientate the GRF vector and to reduce the loads in the affected area of the joint while walking [43–45]. Previous studies have shown that shifting the posterior pod laterally causes a reduction in the knee adduction moment, which is an indicator for knee OA severity, and alters muscle activation patterns. In addition, the convex nature of the elements induces a level of controlled perturbation and proprioceptive training causing muscles in the lower limb to work differently [43–47]. The combination of altered forces and moments acting on the affected joint as a result of the device set-up, combined with controlled perturbation allows a neuromuscular training response to occur [38, 48].

The treatment is undertaken whilst going about daily activities to induce multiple functional repetitions and promotes implementation of new motor patterns and better neuromuscular control. Patients with knee OA that have been previously treated with this biomechanical device have shown reductions in knee adduction and flexion moments during barefoot walking, which were accompanied by significant reductions in pain and subsequent improvements in function and quality of life [31, 38, 48]. Previous studies have also suggested a positive clinical effect for the device when addressing other musculoskeletal conditions such as degenerative meniscal tear, post total knee replacement, hip OA, post total hip replacement and in patients with chronic non-specific low back pain [49–54].

All patients received instructions to wear the biomechanical devices during walking in his/her daily routine (at home/work), for half an hour a day at the start of the treatment, gradually increasing (+ 10 min/week) up to 3 h/day after 3 months. Patients undertook follow-up assessment and re-calibration of the device approximately 3, 12, and 24 weeks after commencing the treatment in-order to optimise the pain alleviating characteristics of the device and progress the convexity to induce further neuromuscular challenges as required. Typically, patients commence treatment whilst presenting moderate-severe symptoms and often in the advanced stages of the disease. The intervention is often sought after they have explored other non-surgical treatment modalities such as traditional physiotherapy, knee joint injections and other non-invasive treatments. Despite the apparent ineffectiveness of these treatment modalities for the patient to this point, patients are often reticent to abandon traditional care measures currently in place. To ensure compliance with the current study, patients were allowed to continue with traditional care in conjunction to the intervention, as they saw fit.

### Statistical analysis

Data was analysed with SPSS software version 23.0 with significance levels set at *p* < 0.05. Data is presented as mean and standard deviations for gait spatial-temporal parameters and questionnaires (WOMAC/SF-36), followed by a 95% confidence interval (CI) for all time periods. The Kolmogorov-Smirnov non-parametric test was used to establish the normal distribution of spatial-temporal parameters and therefore use parametric statistical tests. To assess for the potentially confounding effects of age and gender in the WOMAC and SF-36 scores, analysis of covariance was conducted.

WOMAC and SF-36 were compared using the non-parametric equivalent methods of Kruskal–Wallis test to compare all three groups, and Mann–Whitney U-tests for pairwise comparisons. Repeated measure ANOVA’s were performed for the differences between the outcomes, measured at baseline, 3 and 6 months into treatment, as well as sub-group analysis of patients that were recommended surgery and patients that were not recommended surgery. Multiple linear regression analysis was used to address the percentages changes after 6 months. The WOMAC and SF-36 scores were the dependent variables, with the gait parameters used as independent variables. Because age is typically related to PCS and gender to MCS, the linear regression models initially included age and gender. Interaction terms were also included in the regression model. To adjust for the potentially confounding effects of socio-demographic factors (age and sex) in the SF-36 and WOMAC scores, analysis of covariance was conducted. All reported background values are from two-sided tests.

In addition, we calculated the Outcome Measures in Rheumatology Clinical Trials (OMERACT)-Osteoarthritis Research Society International (OARSI) responder criteria for clinically significant improvement for each of the patients [55]. These stipulate either an improvement in total, pain or in function WOMAC sub-scales of at least 50% with a decrease of 2 cm on the visual analogue-scale for pain or function, or an improvement in both pain and function of at least 20% with a decrease of 1 cm on the VAS [55].

The correlation between gait changes and questionnaire improvement was calculated (i.e. the difference between baseline and 6 months).

KOFG descriptive statistics was calculated to reflect changes over time. KOFG is a categorical data and was analysed using Chi-square test to compare proportions of various scores of the study.

## Results

All spatial-temporal gait parameters significantly improved following 3 months of treatment (all less than *p* < 0.01). There were also further significant improvements in all parameters between 3 and 6 months of treatment (All less than *p* < 0.01), except SLS on both sides (*p* = 0.554 and 0.452). After 6 months of treatment, all parameters significantly improved compared to baseline. Specifically, gait velocity, step length and SLS of the more symptomatic knee improved by 13, 7.8 and 3% respectively (*p* < 0.01). The changes in gait over the time intervals are summarised in Table 2.

**Table 2:** Spatial-temporal parameter changes in knee OA patients after 3 and 6 months of treatment. Results are presented as mean (SD) [95% CI]

	Baseline	3 months	6 months	*P*-value
Velocity (cm/s)	91.88 (20.68) [89.97–93.78]	102.69 (19.19) [100.92–104.46]	104 (19.12) [102.23–105.76]	< 0.001
Step length- more symptomatic limb (cm)	53.96 (8.8) [53.15–54.78]	57.70 (8.36) [56.93–58.48]	58.15 (8.36) [57.384–58.93]	0.001
Step length- less symptomatic limb (cm)	54.07 (9.0) [53.24–54.9]	57.86 (8.63) [57.06–58.66]	58.34 (8.44) [57.564–59.12]	< 0.001
Single limb support- more symptomatic limb (% GC^*^)	36.55 (2.42) [36.32–36.79]	37.68 (2.10) [37.49–37.88]	37.72 (2.09) [37.53–37.92]	< 0.001
Single limb support- less symptomatic limb (% GC^*^)	38.34 (2.06) [38.15–38.53]	38.53 (1.99) [38.34–38.71]	38.56 (1.93) [38.39–38.74]	0.003

*N* = 455; *GC Gait Cycle; *P*-value was set to *P* < 0.05

Repeated measure ANOVA’s were performed for the differences between the outcomes, measured at baseline, 3 and 6 months into treatment. Significant differences were found between baseline and 3 months and baseline and 6 months

There was a significant improvement in KOFG between baseline and 3 months follow-up (*p* < 0.001), with retained improvements at 6 months. Table 3 displays the changes in classification over the 6 months of treatment. It shows a shift from more severe functional grades to less severe levels over time (improved functional outcomes). More specifically, at baseline two thirds (71%) of the patients were classified with grade 1 and 2 (i.e. mild-moderate functional limitation) and a third of the patients (29%) were classified with grade 3 and 4 (i.e. moderate-severe functional severity). After 6 months of treatment 86% of the patients were with a functional classification grade 1 & 2 and 14% with grade 3 and 4 respectively.

**Table 3:** Knee Osteoarthritis Functional Grade (KOFG)^*^ changes in patients after 3 and 6 months of treatment. Values are presented as n (%)

	Baseline	3 months	6 months
Grade 1	117 (25.71%)	190 (41.76%)	197 (43.29%)
Grade 2	207 (45.49%)	193 (42.42%)	196 (43.07%)
Grade 3	95 (20.88%)	62 (13.63%)	52 (11.43%)
Grade 4	36 (7.91%)	10 (2.19%)	10 (2.19%)

Descriptive statistics of patient’s grade distribution over time. The average grade was 2.1 at baseline, 1.8 at 3 months and 1.7 at 6 months. Significant differences were found in paired T-test between baseline and 3 months (*p* < 0.001) and between baseline and 6 months (p < 0.001). There were no significant differences in mean grade score between 3 and 6 months (*p* = 0.01) *Grade 1- least severe, Grade 4- most severe

Following 6 months of treatment, all patients’ self-evaluation questionnaires improved significantly. All WOMAC subscales significantly improved following 3 months of treatment, with further improvements at 6 months (*p* < 0.001). WOMAC Total, along with pain, function and stiffness subscales improved by 46.2, 48.6, 45.7 and 43.4% respectively (*p* < 0.001 for all). 67% of the patients met the OMERACT-OARSI criteria (307/455 significantly improved). Table 4 displays the absolute changes from baseline to 6 months.

**Table 4:** Patient reported outcome measures (PROM’s) changes in knee OA patients after 3 and 6 months of treatment. Results are presented as mean (SD) [95% CI]

	Baseline	3 months	6 months	*P*-value^*^
WOMAC^**^				
Total	41.40 (19.68) [39.58–43.21]	25.32 (19.13) [23.56–27.08]	22.28 (18.13) [20.61–23.96]	< 0.001
Pain subscale	46.71 (18.86) [44.97–48.44]	27.42 (19.72) [25.60–29.24]	23.99 (18.94) [22.25–25.74]	< 0.001
Function subscale	38.97 (21.56) [36.98–40.96]	24.07 (19.78) [22.25–25.9]	21.16 (18.50) [19.45–22.86]	< 0.001
Stiffness subscale	48.74 (25.92) [46.35–51.13]	30.71 (25.92) [28.48–32.95]	27.59 (23.15) [25.45–29.72]	< 0.001
SF-36^***^				
Total	53.49 (16.08) [52.01–54.97]	62.60 (16.57) [61.08–64.13]	65.22 (16.87) [63.67–66.78]	< 0.001
Physical Component Summary (PCS)	45.67 (18.36) [43.98–47.36]	57.71 (19.85) [55.88–59.54]	61.37 (20.04) [59.52–63.21]	< 0.001
Mental Component Summary (MCS)	64.02 (19.49) [62.23–65.82]	72.30 (18.18) [70.63–73.98]	73.64 (18.12) [71.97–75.31]	< 0.001

*P-value was set to *P* < 0.05. **Western Ontario and McMaster Universities Index (WOMAC Index). The WOMAC questionnaire includes 24 questions in a Visual Analogue Scale (VAS) format (0 = no pain/stiffness/difficulty, 100 = severe pain/stiffness/difficulty). ***SF-36 Health Survey includes 36 questions. Results range between 0 and 100 (0 = poor quality of life, 100 = high quality of life)

Repeated measure ANOVA’s were performed for the differences between the outcomes, measured at baseline, 3 and 6 months into treatment. Significant differences were found between baseline and 3 months and baseline and 6 months

All SF-36 subscales also significantly improved following 3 months of treatment (*p* < 0.001). There were also further significant improvements between 3- and 6-month time intervals, except for the MCS (*p* = 0.068). After 6 months of treatment all subscales had significantly improved (*p* < 0.001). Specifically, SF-36 Total, PCS and MCS improved by 11.73, 15.7, and 9.62 points, or 22, 34 and 15% respectively compared to baseline (See Table 4). These improvements also met minimal clinical important differences (MCID) for clinical significance of 7.8 points [56].

A correlation analysis between gait and questionnaire improvement was calculated. First, a new parameter was calculated for each measurement. This was the difference between baseline and 6 months. Second, a correlation calculation between the changes in gait measures and the changes in questionnaires was calculated. A significant correlation was found between the changes in gait parameters and the changes in questionnaires (*p* < 0.05 for all). More specifically, the correlation between the changes in gait velocity and the changes in pain, function, PCS and MCS was − 0.30, − 0.29, 0.33 and 0.20 respectively.

**Fig. 3:**
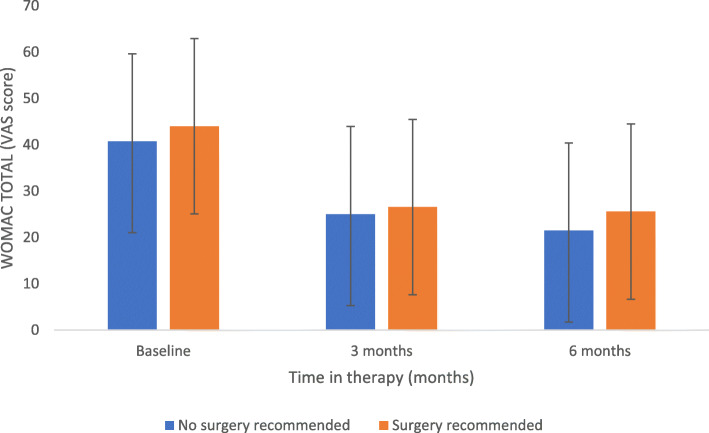
Comparison of WOMAC changes between patients that had been recommended for knee joint replacement surgery (Total Knee Replacement/Partial Knee Replacement, TKR/PKR respectively) and patients that were not

A sub-analysis was carried out on patients that had already been recommended knee joint replacement surgery (surgery recommended group) prior to commencing treatment (20%, 92 patients). There were no significant differences in any baseline measures (WOMAC, SF-36 or gait parameters) between this cohort and the rest of the patients, apart from duration of symptoms prior to commencing treatment (no surgery recommended group: 67 months vs surgery recommended group: 83 months, *p* = 0.013). Both groups improved significantly over time (*p* < 0.05), meeting the MCID in all outcome measures. Despite improvements in all variables of both groups at 6 months, the recommended surgery group displayed higher WOMAC pain and stiffness subscales (*p* = 0.027, and *p* = 0.019, see Fig. 3), and lower SLS in both the more and less symptomatic sides (*p* = 0.04 and *p* = 0.028).

## Discussion

The purpose of this study was to investigate the changes in spatial-temporal gait parameters and clinical measurements following treatment with a non-invasive foot-worn biomechanical device on patients with knee osteoarthritis within the UK. Following 3 months of treatment, patients demonstrated significant improvements in both gait and PROM’s, with maintained or further improvements occurring in all parameters after 6 months. The results suggest that most improvements occurred by 3 months, but further improvements occur between 3 and 6 months, apart from SLS in gait and MCS subscale of SF-36. This supports previous work which found that the largest improvements occurred within the initial few months of treatment and are then maintained [32]. The improvements in WOMAC scores meet the OMERACT-OARSI guidelines for clinical response to treatment in 67% of the patients, signifying true positive impacts felt by patients [55]. The thresholds for minimal clinical importance differences (MCID) within SF-36 were also met, suggesting improvements in quality of life following treatment [56]. In addition, these improvements in self-evaluation questionnaires correlated with the significant improvements in gait.

PROM’s along-with radiographic findings have historically been used to track knee OA outcomes and are leading predictors in guiding the decision upon joint replacement surgery [57]. However, the low correlation between radiographic findings and patient symptoms has since become more acknowledged [58], and therefore the importance of more objective and functional measures to evaluate patient symptoms has become apparent. Previous research has proposed spatial-temporal parameters are a good indicator for functional severity [12, 58], with a recent meta-analysis suggesting that stride duration and cadence provided a better reflection of knee OA severity than kinematic and kinetic measures [59].

Using the patients’ cadence and stride lengths as predictor variables for knee OA severity forms the basis of the validated Knee Osteoarthritis Functional Grade or KOFG [13], which in a follow-up study was also validated as a classification tool to measure treatment effect [60]. A combination of spatial-temporal parameters objectively classifies patients with knee OA according to functional disease severity, which have been shown to correlate with radiographic evaluation, the level of pain, function and rate of TKR. The benefit of this tool is the ability to quantify the severity of disease and to assess the impact of an intervention, rather than just stating the change in gait analysis parameters [60]. The model suggests that a shorter stride length with lower cadence is indicative of a higher functional severity grade (more severe knee OA), while a longer stride length with higher cadence is indicative of a lower functional severity grade (less severe knee OA). Within this current study, there was a significant improvement in KOFG between baseline and 3 months follow-up (*p* < 0.001), with retained improvement at 6 months. The shift from more severe to less severe KOFG suggest patients not only improvement in symptoms, but actually move away from measures associated with increased rates of TKR [13, 60].

The present study also examined whether there were any differences in the sub-group of patients (20%) that had already been offered joint replacement surgery (TKR/PKR) prior to commencing treatment. Interestingly, aside from the duration of symptoms, there were no significant differences between cohorts at baseline, suggesting that those patients that had been recommended surgery as a suitable option for their condition, displayed the same characteristics as those that have not had surgical recommendation (Fig. 3). Research reports most patients that are suitable for TKR have WOMAC baseline scores between 40s to 50’s [57], which could indicate that the cohorts analysed within this study are representative of this population. Furthermore, significant improvements seen across both groups (Fig. 3) suggests these populations can respond well to this treatment in levels of pain, function and quality of life measures. Differences between these two groups were seen at 6 months, including significantly higher WOMAC pain and stiffness subscales (*p* = 0.027, and *p* = 0.019), and significantly lower SLS in both the more and less symptomatic sides (*p* = 0.04 and *p* = 0.028) in the recommended surgery group. The reduction of improvements within this group could be explained by significantly longer durations of symptoms experienced by the patients, suggesting more chronicity of the condition. Previous studies have investigated the durations of symptoms and their relationship to clinical improvements and have suggested that optimising the timing that patients access relevant treatments could be key to optimising outcomes [61]. Despite this, the overall improvements in both groups are marked and suggestive that the treatment could be an effective alternative for a number of patients that would otherwise have progressed to surgery.

Researchers have presented several theories explaining how this treatment works to improve symptoms in patients with knee OA. Studies have shown that the biomechanical device can reduce the external loads acting on the body to “unload” the painful area, which is said to be important when treating this condition [43, 44]. It has been shown to reduce the 1st and 2nd peak KAM and KAAI by 8.4, 12.7 and 13% after 9 months of treatment respectively [38]. An important factor to note with the improvements in biomechanical variables seen with the device is that they remained, even when the patient was not wearing the device. This suggests that a motor learning effect occurs as a result of the neuromuscular retraining received from the treatment [46]. Neuromuscular training is delivered by a controlled level of perturbation via the convex nature of the elements [46, 47]. The combined features of the biomechanical device allow for repetitive perturbations with diminished pain throughout the gait cycle. Patients wearing the devices for prescribed periods every day means that they gain high repetitions of closed kinetic chain, functional exercises and improved levels of compliance said to be advantageous for motor learning [62–64]. This combination of key rehabilitation principles allows the patient to reacquire improved neuromuscular control, thus avoiding pathological patterns previously utilised whilst in pain [31].

Given knee OA is a chronic degenerative condition, symptoms deteriorate over time at varying rates [65]. Currently, interventions are directed to the end-stages of the disease and therefore can often be ineffective and palliative in nature [66]. Whilst the literature reports that the number of patients progressing to having joint replacement surgery is growing [6], approximately 20–40% of those completed are considered inappropriate. These were classified due to having only slight or moderate symptoms, or not severe enough radiographic findings and therefore TKR deemed unnecessary [67, 68]. A paradigm shift is needed to focus efforts on treating patients at high-risk earlier in the disease progression [66], or utilising more specialist modalities that can help minimise this potentially inappropriate flow to surgery. The results of this study display a combination of improvements in both subjective PROM’s and objective spatial-temporal gait parameters which are promising and may indicate that the biomechanical device used in this study has the ability to be an effective modality in managing this chronic condition, however further studies in a controlled setting are required.

This study has some limitations. Firstly, the study was a retrospective analysis of patients from the centres database and therefore had no control group. However, a previous study has demonstrated comparable positive effects of this treatment compared to a control group in a double-blind study [31]. In addition, patients were allowed to continue with traditional care, and we cannot determine that other treatment did not affect the results of this study. Patients are usually characterised with a moderate-severe knee OA and commence the current treatment after trying traditional care with little to no success. The treatment is often undertaken as one final attempt to address the condition non-invasively prior to the need for a surgical intervention. As a result, we believe most of the clinical effect seen in this study can be attributed to the biomechanical device and treatment plan as opposed to any adjunctive or continued treatment modalities. Potentially, a combined approach of exercise therapy utilised alongside the biomechanical treatment may yield further superior effects compared to the device alone and this should be investigated in a controlled setting in the future.

Secondly, this study had a relatively short follow-up duration of 6 months for this cohort. Long-term follow-up would give more insight into the lasting effects of the treatment. However, it reflects previous research on the treatment on different populations with similar improvements in gait and PROM’s [33–35, 69]. Therefore, it could be assumed that the improvements can be maintained with the high compliance rates in the treatment [32]. Nevertheless, future research should continue to investigate the long-term clinical effect of the treatment, in prospective, randomised control trial (RCT) design whilst tracking decay rates for joint replacement surgeries. Promisingly, preliminary data from an RCT on the effect of this treatment displays comparable improvements to this study [70].

Lastly, this study did not monitor the overall activity level of the patients in general and this compliance to the treatment plan in specifics. We cannot confirm the usage time of the device at home other than when the patients returned to the clinic for a follow-up appointment and reported that they have been using the device daily. Future studies should enforce methods to monitor compliance to the treatment plan at home.

## Conclusions

The examined non-invasive biomechanical treatment led to a significant improvement in gait patterns, pain, function and quality of life for UK patients suffering with knee OA, although further studies in a controlled setting are required to investigate its clinical effect further. It appears to create a comparable response between patients that have already been recommended knee joint replacement surgery and those that have not been recommended, therefore potentially providing an alternative solution for this population. If these results can be retained in the longer term, it could hypothetically delay or even avoid the need for surgery in many cases which provides an area for examination in future trials. Whilst further studies in controlled settings are required in order to fully understand the clinical effect of this treatment, this study suggests that it may provide positive clinical implications for patients, with the potential to provide a sustainable modality for healthcare systems to manage knee OA patients effectively in the community setting.

## Data Availability

All data requests will be reviewed and addressed by the authors.

## Abbreviations

OA: Osteoarthritis
NICE: National Institute of Clinical Excellence (NICE)
TKR: Total knee replacement (TKR)
PROM’s: Patient reported outcome measures (PROM’s)
KAM: Knee adductor moment (KAM)
KAAI: Knee adductor angular impulse (KAAI)
LED: Light emitting diode (LED)
SLS: Single-limb support (SLS)
KOFG: Knee Osteoarthritis Function Grade (KOFG)
VAS: Visual analogue scale (VAS)
PCS: Physical Component Summary (PCS)
PKR: Partial Knee Replacement
MCS: Mental Component Summary (MCS)
COP: Center of pressure (COP)
OMERACT-OARSI: Outcome Measures in Rheumatology Clinical Trials (OMERACT)-Osteoarthritis Research Society International (OARSI)
